# Depressive disorders, bad mental health days, and diabetes management behaviors among non-Hispanic American Indian/Alaska Native adults: Findings from the Behavioral Risk Factor Surveillance System

**DOI:** 10.1371/journal.pone.0327870

**Published:** 2025-07-14

**Authors:** Kaipeng Wang, Luohua Jiang, Jie Zhu, Spero M. Manson

**Affiliations:** 1 Graduate School of Social Work, University of Denver, Denver, Colorado,; 2 Joe C. Wen School of Population and Public Health, University of California Irvine, Irvine, CA,; 3 School of Family and Consumer Sciences, Texas State University, San Marcos, Texas,; 4 Centers for American Indian and Alaska Native Health, University of Colorado Anschutz Medical Campus, Aurora, Colorado; University of New Mexico Health Sciences Center, UNITED STATES OF AMERICA

## Abstract

**Objective:**

This study examined the association between diagnosis of depressive disorder, the number of bad mental health days per month, and diabetes management behaviors among American Indian/Alaska Native (AI/AN) adults with diabetes.

**Research design and methods:**

Data were drawn from the Behavioral Risk Factor Surveillance System (2018–2021), including 2,272 self-identified non-Hispanic AI/AN adults diagnosed with non-gestational diabetes. Key variables included a self-reported prior diagnosis of depressive disorder and the number of bad mental health days in the past month. Outcome variables were seven diabetes management behaviors, such as taking a diabetes management class and performing daily foot checks. Statistical analyses included descriptive statistics, chi-squared tests, ANOVA, and logistic regression models.

**Results:**

Among the participants, 24.8% were diagnosed with depressive disorder, and 19.5% reported at least 14 bad mental health days in the past month. Logistic regression models show that those reporting depressive disorders were significantly less likely to check their feet daily (adjusted odds ratio (AOR) = 0.56, 95% CI: 0.34–0.92). Individuals with at least 14 bad mental health days were significantly less likely to have ever taken a diabetes management class (AOR = 0.59, 95% CI: 0.36–0.99) and check their feet daily (AOR = 0.37, 95% CI: 0.21–0.65) than those reporting no bad mental health days.

**Conclusions:**

Depressive disorders and frequent bad mental health days were associated with lower odds of diabetes management behaviors among AI/AN adults. These findings suggest that enhancing mental health support within diabetes management programs may help address disparities in diabetes care among AI/AN adults.

## Introduction

Diabetes is a challenging chronic health condition that affects the daily activities and quality of life of over half a billion people in the world [1]. The United States is not immune to the worldwide epidemic of diabetes. The American Diabetes Association (ADA) estimated that about 11.3% of the U.S. population reported having been diagnosed with diabetes, which represents over 37 million Americans [2]. In addition to the high prevalence of diabetes in the general population, a salient disparity gap exists across racial groups. Specifically, American Indians/Alaska Natives (AI/AN) report the highest age-adjusted prevalence of diabetes (~15%), reflecting twice the rate among non-Hispanic White Americans [3]. Furthermore, AI/AN individuals living with diabetes (ILDs) are significantly more likely to die from diabetes-related complications than other racial/ethnic groups [4].

To address these striking disparities, the Indian Health Service (IHS) has made substantial strides over the past few decades, such as managing the Special Diabetes Program for Indians [5–7]. Those efforts have led to significant improvements in the prevention and management of diabetes among AI/AN populations, including reduced rates of diagnosed diabetes, severe hypoglycemia, severe hyperglycemia, and diabetes-related end-stage renal disease [5–7]. Nonetheless, other diabetes-related disparities, such as cardiovascular disease, depression, and anxiety, remain profound in the AI/AN population [8,9]. These enduring disparities underscore the need for continued, focused efforts to bridge the gap in diabetes care and outcomes for AI/AN communities.

The adverse consequences of diabetes have been well-established by a compelling body of literature. Epidemiological studies revealed that ILDs are 2–4 times more likely to develop cardiovascular diseases than individuals without diabetes, and over half of the ILDs will develop secondary chronic conditions, like peripheral neuropathy or nephropathy [10–12]. Additionally, ILDs consistently report significantly higher risks of comorbid mental health challenges, compromised quality of life, and poor general wellness [13–16]. AI/AN ILDs, unfortunately, are even more susceptible to many negative consequences of diabetes. The American College of Cardiology revealed that AI/AN ILDs are at least twice more likely to develop coronary heart disease than their non-Hispanic white counterparts [17]. Similarly, AI/AN ILDs report significantly lower overall quality of life than ILDs from other racial groups [18], emphasizing the need for comprehensive and targeted interventions to address these disparities.

There is ample existing evidence showing the effectiveness of diabetes management in promoting ILDs’ quality of life, especially by improving ILDs’ self-management behaviors [19–21]. Kent and colleagues, for example, revealed that improved diabetes self-management is associated with reduced risks of diabetes complications [22]. Similarly, Rasoul and colleagues reported that effective self-management behaviors significantly improve ILDs’ biomedical parameters of diabetes severity (BMI, HbA_1c_) and overall quality of life [23]. However, it has also been shown that mental health factors can negatively affect ILDs’ diabetes management behaviors, especially depression. Diabetes poses high levels of stress on ILDs’ mental health status, while compromised mental health is closely connected with interrupted and suboptimal diabetes self-management behaviors (19). One in four people with type 2 diabetes reports clinically significant depression, and depression is associated with worse glycemic control and poorer management of diabetes (28–32).

Despite extensive literature documenting the association between depression and diabetes self-management behaviors, research on this relationship among AI/AN ILDs is limited. A systematic review of the emotional and behavioral aspects of diabetes in AI/AN individuals identified only nine studies that focused on depression, none of which focused on the relationship between depression and self-management behaviors [24]. In addition, the relationship between self-reported mental health, such as the frequency of bad mental health days and diabetes management is also under-studied in this population. Diabetes management requires a diligent and consistent approach to reinforcing medication adherence, regular blood glucose monitoring, dietary management, and physical activity, all of which can be significantly influenced by an individual’s mental health status. Poor mental health, manifesting as increased bad mental health days, can exacerbate the challenges of managing diabetes by reducing an individual’s motivation, energy, and cognitive capacity and increasing emotional distress to adhere to these necessary routines [25]. Therefore, this study examined associations among self-reported prior diagnosis of depressive disorder, number of bad mental health days, and diabetes management behaviors among AI/AN ILDs.

## Materials and methods

### Study design

The data for this study were drawn from the Behavioral Risk Factor Surveillance System (BRFSS), which is an ongoing health survey system established by the Centers for Disease Control and Prevention (CDC) to describe chronic health conditions and health service use, as well as related protective and risk factors among noninstitutionalized adults aged 18 or older [26]. BRFSS data are acquired by both landline telephone and cellular telephone-based surveys. Since this study is secondary research using publicly available data and does not require consent, the University of Denver’s Institutional Review Board (IRB) has classified it as exempt from review under federal regulations (IRB Number: 2182978−1). Data were accessed on April 12, 2024, for research purposes. The authors do not have access to information that could identify individual participants during or after data collection.

We pooled data from Waves 2018, 2019, 2020, and 2021, which are the most recent waves of data that contain comprehensive information on diabetes management behaviors related to this study. The combined dataset included responses from 1,696,355 participants aged 18 and older. Participants were included in the analytic sample if they (1) reported having been told by a healthcare provider that they had diabetes and (2) self-identified as non-Hispanic American Indian or Alaska Native only. Participants were excluded if their diabetes was limited to gestational diabetes (i.e., diagnosed only during pregnancy). After applying these criteria, the final analytic sample consisted of 2,272 self-identified non-Hispanic AI/AN adults with a self-reported prior diagnosis of non-gestational diabetes.

### Measures

#### Outcome variables.

The outcome variables were seven self-reported diabetes management behaviors. Consistent with the literature [27], diabetes management behaviors for the current study included (1) ever attending a diabetes management class (yes vs. no), (2) foot self-check for sores or irritation (at least once per day vs. less than once per day), (3) glucose self-check (at least once per day vs. less than once per day), (4) biannual diabetes clinical visit (at least twice per year vs. less than twice per year), (5) biannual HbA_1c_ check (at least twice per year vs. less than twice per year), (6) annual foot check by a professional (at least once per year vs. less than once per year), and (7) annual dilated eye examination (at least once per year vs. less than once per year).

#### Key exposure variables.

Key exposure variables included self-reported prior diagnosis of depressive disorder and self-reported number of bad mental health days per month. For self-reported prior diagnosis of depressive disorder, participants were asked whether they were ever told that they had a depressive disorder, including depression, major depression, dysthymia, or minor depression. Self-reported number of bad mental health days per month was ascertained by asking participants “thinking about your mental health, which includes stress, depression, and problems with emotions, for how many days during the past 30 days was your mental health not good?” Responses, consistent with the BRFSS study design and previous literature [28,29], were recoded into three categories: (1) 0 day (reference group), (2) 1–13 days, and (3) at least 14 days.

#### Covariates.

Covariates were selected according to the fundamental cause theory of diseases, which emphasized the importance of both individual and contextual factors as causes of health disparities [30], as well as the existing literature on diabetes management [27]. Covariates for the current study included age (younger adults: 19–44 years; middle-aged adults: 45–64 years; older adults: 65 or older), age of diabetes diagnosis in years, sex (biological sex assigned at birth: male vs female), marital status (not married/partnered vs married/partnered), employment status (not employed vs employed), annual household income (less than $15,000, $15,000 to less than $25,000, $25,000 to less than $35,000, $35,000 to less than $50,000, and $50,000 or more), urban-rural status (living in an urban county vs living in a rural county), access to health insurance (having some form of insurance vs not having any form of health insurance), access to health care providers (having at least one personal health care provider vs not having any personal health care provider), and survey wave (2018, 2019, 2020, and 2021).

### Statistical analysis

All statistical analyses were conducted using Stata MP 18 (StataCorp LLC, College Station, Texas). Two-sided p-values less than 0.05 were considered statistically significant. We report descriptive statistics, including (1) mean and standard deviation for age when diagnosed with diabetes and (2) percentage for all the other variables, to summarize sample characteristics for the whole analytical sample, by self-reported prior diagnosis of depressive disorder, and by number of bad mental health days per month. We used chi-squared tests and independent samples t-tests to compare the sample characteristics by self-reported prior diagnosis of depressive disorder. We used chi-squared tests and one-way Analysis of Variance (ANOVA) to compare the sample characteristics by number of bad mental health days.

After applying complex sampling weights, logistic regression models were used to examine the association of self-reported prior diagnosis of depressive disorder and number of bad mental health days with diabetes management behaviors after controlling for covariates. Previous research has shown that mental health is associated with diabetes management education [31]. It is important to consider the potential mediation effect of ever taking diabetes management class for the other diabetes management behaviors. Thus, four models were estimated for each diabetes management behavioral outcome: (1) Model 1 regressed the outcome on self-reported prior diagnosis of depressive disorder after controlling for all covariates; (2) Model 2 added ever taking a diabetes management class as a covariate based on Model 1 for all outcomes except for ever taking a diabetes management class; (3) Model 3 regressed the outcome on number of bad mental health days per month after controlling for all covariates; (4) Model 4 added ever taking a diabetes management class as a covariate based on Model 3 except for ever taking a diabetes management class. The percentage of missing values was highest for income (17.1%), whereas this was no greater than 6.7% for all other variables in the current study. To deal with missing values, we estimated the above-mentioned models using both listwise deletion of missing values and multiple imputation by chained equations (MICE) to ensure transparency and assess robustness of findings, as recommended by previous literature [32].

### Data and resource availability

The datasets generated during and/or analyzed in the current study are available on the BRFSS website: https://www.cdc.gov/brfss/annual_data/annual_data.htm.

## Results

Table 1 summarizes the sample characteristics. To highlight a few of the key variables of the study, 24.8% of participants were diagnosed with depressive disorder. While 59.0% reported no bad mental health days in the past month, 21.6% reported having 1–13 bad mental health days, and 19.5% reported at least 14 bad mental health days in the past month. 56.9% of participants reported having taken a diabetes management class. For self-management behaviors, 67.3% checked their feet at least once per day and 67.1% checked their glucose at least once per day. Moreover, the percentages of at least biannual clinical visits for diabetes and biannual HbA_1c_ test were respectively 73.9% and 70.0%. The percentages of at least annual professional foot check, and dilated eye exam were respectively 76.5% and 69.4%.

**Table 1:** Characteristics of the non-Hispanic AI/AN participants with a self-reported diagnosis of non-gestational diabetes in BRFSS for the whole sample including those with and without non-gestational diabetes, 2018-2021 (N = 2,272).

Variables	Frequency (%) or Mean (±SD)	Missing values N (%)
ever took diabetes management class		9 (0.4%)
no	976 (43.1%)	
yes	1,287 (56.9%)	
foot self-check at least once per day		68 (3.0%)
no	721 (32.7%)	
yes	1,483 (67.3%)	
glucose self-check at least once per day		62 (2.7%)
no	728 (32.9%)	
yes	1,482 (67.1%)	
biannual clinical visit for diabetes		79 (3.5%)
no	572 (26.1%)	
yes	1,621 (73.9%)	
biannual HbA_1c_ test		125 (5.5%)
no	645 (30.0%)	
yes	1,502 (70.0%)	
annual foot check by a professional		75 (3.3%)
no	517 (23.5%)	
yes	1,680 (76.5%)	
annual dilated eye exam		53 (2.3%)
no	679 (30.6%)	
yes	1,540 (69.4%)	
self-reported prior diagnosis of depressive disorder		14 (0.6%)
no	1,698 (75.2%)	
yes	560 (24.8%)	
number of bad mental health days per month		69 (3.0%)
none	1,299 (59.0%)	
1-13 days	475 (21.6%)	
14 + days	429 (19.5%)	
education level		10 (0.4%)
below high school	334 (14.8%)	
high school or GED	719 (31.8%)	
some college	750 (33.2%)	
college or above	459 (20.3%)	
age		0 (0%)
19–44 years	283 (12.5%)	
45–64 years	1,150 (50.6%)	
65 or older	839 (36.9%)	
sex		0 (0%)
male	970 (42.7%)	
female	1,302 (57.3%)	
marital status		11 (0.5%)
not married/partnered	1,369 (60.5%)	
married/partnered	892 (39.5%)	
employment status		32 (1.4%)
not employed	1,463 (65.3%)	
employed	777 (34.7%)	
income		389 (17.1%)
less than $15,000	460 (24.4%)	
$15,000 to less than $25000	507 (26.9%)	
$25,000 to less than $35,000	267 (14.2%)	
$35,000 to less than $50,000	229 (12.2%)	
$50,000 or more	420 (22.3%)	
urban-rural status		1 (<0.1%)
urban	1,152 (50.7%)	
rural	1,119 (49.3%)	
age of diabetes diagnosis	45.250 (±14.294)	152 (6.7%)
access to health insurance		31 (1.4%)
no health insurance	122 (5.4%)	
having health insurance	2,119 (94.6%)	
access to health providers		19 (0.8%)
no health provider	349 (15.5%)	
at least one health provider	1,904 (84.5%)	
wave		0 (0%)
2018	602 (26.5%)	
2019	552 (24.3%)	
2020	405 (17.8%)	
2021	713 (31.4%)	

Table 2 compares the sample characteristics by self-reported prior diagnosis of depressive disorder and number of bad mental health days per month. Among all diabetes management outcomes, only annual dilated eye exams significantly differed between those who were diagnosed with depressive disorder and those who were not. While 70.6% of those who were not diagnosed with a depressive disorder completed annual dilated eye exam, 65.9% of those diagnosed with depressive disorders had an annual dilated eye exam (p = 0.039). The number of bad mental health days per month, age, sex, employment status, income, urban-rural status, age at diabetes diagnosis, and access to health providers significantly differed by self-reported prior diagnosis of depressive disorders.

**Table 2:** Characteristics of the AI/AN adults in BRFSS with a self-reported diagnosis of non-gestational diabetes by self-reported prior diagnosis of a depressive disorder and number of bad mental health days per month, 2018-2021.

	self-reported prior diagnosis of depressive disorder			number of bad mental health days per month			
	no	yes	P	none	1-13 days	14 + days	P
N	1,698 (75.2%)	560 (24.8%)		1,299 (59.0%)	475 (21.6%)	429 (19.5%)	
ever took diabetes management class							
no	720 (42.5%)	248 (44.5%)	0.403	531 (41.0%)	201 (42.6%)	207 (48.3%)	0.032
yes	974 (57.5%)	309 (55.5%)		763 (59.0%)	271 (57.4%)	222 (51.7%)	
foot self-check once a day							
no	543 (32.9%)	172 (31.7%)	0.607	394 (31.3%)	167 (35.9%)	129 (30.9%)	0.154
yes	1,106 (67.1%)	370 (68.3%)		866 (68.7%)	298 (64.1%)	288 (69.1%)	
glucose self-check once a day							
no	541 (32.8%)	181 (33.0%)	0.958	396 (31.6%)	163 (35.1%)	138 (32.5%)	0.393
yes	1,106 (67.2%)	368 (67.0%)		858 (68.4%)	302 (64.9%)	286 (67.5%)	
biannual clinical visit for diabetes							
no	433 (26.5%)	138 (25.2%)	0.544	328 (26.2%)	109 (23.5%)	114 (27.4%)	0.375
yes	1,201 (73.5%)	410 (74.8%)		923 (73.8%)	355 (76.5%)	302 (72.6%)	
biannual HbA_1c_ test							
no	477 (29.8%)	166 (31.1%)	0.579	352 (28.5%)	132 (29.3%)	135 (33.4%)	0.175
yes	1,123 (70.2%)	368 (68.9%)		881 (71.5%)	318 (70.7%)	269 (66.6%)	
annual foot check by a professional							
no	372 (22.6%)	139 (25.6%)	0.158	271 (21.6%)	105 (22.7%)	119 (28.4%)	0.016
yes	1,271 (77.4%)	404 (74.4%)		984 (78.4%)	357 (77.3%)	300 (71.6%)	
annual dilated eye exam							
no	489 (29.4%)	186 (34.1%)	0.039	364 (28.6%)	138 (29.7%)	158 (38.0%)	<0.001
yes	1,172 (70.6%)	359 (65.9%)		910 (71.4%)	326 (70.3%)	258 (62.0%)	
education level							
below high school	240 (14.2%)	90 (16.1%)	0.519	181 (14.0%)	69 (14.6%)	70 (16.3%)	0.216
high school or GED	532 (31.5%)	181 (32.3%)		407 (31.5%)	140 (29.6%)	148 (34.5%)	
some college	562 (33.3%)	185 (33.0%)		428 (33.1%)	162 (34.2%)	143 (33.3%)	
college or above	354 (21.0%)	104 (18.6%)		276 (21.4%)	102 (21.6%)	68 (15.9%)	
number of bad mental health days per month			<0.001				
none	1,162 (70.4%)	133 (24.7%)					
1-13 days	318 (19.3%)	152 (28.2%)					
14 + days	170 (10.3%)	254 (47.1%)					
self-reported prior diagnosis of depressive disorder							<0.001
no				1,162 (89.7%)	318 (67.7%)	170 (40.1%)	
yes				113 (10.3%)	152 (32.3%)	254 (59.9%)	
age							
19–44 years	183 (10.8%)	98 (17.5%)	<0.001	123 (9.5%)	83 (17.5%)	72 (16.8%)	<0.001
45–64 years	835 (49.2%)	305 (54.5%)		629 (48.4%)	256 (53.9%)	236 (55.0%)	
65 or older	680 (40.0%)	157 (28.0%)		547 (42.1%)	136 (28.6%)	121 (28.2%)	
sex							
male	778 (45.8%)	184 (32.9%)	<0.001	624 (48.0%)	161 (33.9%)	159 (37.1%)	<0.001
female	920 (54.2%)	376 (67.1%)		675 (52.0%)	314 (66.1%)	270 (62.9%)	
marital status							
not married/partnered	1,008 (59.7%)	355 (63.5%)	0.112	758 (58.5%)	287 (60.7%)	281 (66.0%)	0.025
married/partnered	680 (40.3%)	204 (36.5%)		537 (41.5%)	186 (39.3%)	145 (34.0%)	
employment status							
not employed	1,047 (62.5%)	407 (74.0%)	<0.001	818 (63.7%)	296 (63.1%)	297 (70.9%)	0.017
employed	629 (37.5%)	143 (26.0%)		467 (36.3%)	173 (36.9%)	122 (29.1%)	
income							
less than $15,000	325 (23.0%)	132 (28.6%)	<0.001	236 (22.0%)	93 (23.1%)	115 (32.0%)	<0.001
$15,000 to less than $25000	349 (24.8%)	155 (33.5%)		275 (25.7%)	100 (24.8%)	116 (32.3%)	
$25,000 to less than $35,000	201 (14.3%)	63 (13.6%)		161 (15.0%)	56 (13.9%)	44 (12.3%)	
$35,000 to less than $50,000	185 (13.1%)	43 (9.3%)		137 (12.8%)	55 (13.6%)	32 (8.9%)	
$50,000 or more	350 (24.8%)	69 (14.9%)		263 (24.5%)	99 (24.6%)	52 (14.5%)	
urban-rural status							
urban	842 (49.6%)	305 (54.5%)	0.047	643 (49.5%)	249 (52.4%)	221 (51.5%)	0.507
rural	855 (50.4%)	255 (45.5%)		655 (50.5%)	226 (47.6%)	208 (48.5%)	
age of diabetes diagnosis	46.531 (14.313)	41.480 (13.627)	<0.001	46.868 (14.219)	43.349 (13.994)	42.176 (14.314)	<0.001
access to health insurance							
no health insurance	96 (5.7%)	25 (4.5%)	0.275	74 (5.8%)	22 (4.7%)	22 (5.2%)	0.644
having health insurance	1,578 (94.3%)	528 (95.5%)		1,206 (94.2%)	449 (95.3%)	401 (94.8%)	
access to health providers							
no health provider	290 (17.3%)	55 (9.8%)	<0.001	200 (15.5%)	59 (12.5%)	75 (17.6%)	0.098
at least one health provider	1,390 (82.7%)	504 (90.2%)		1,089 (84.5%)	413 (87.5%)	351 (82.4%)	
wave							
2018	456 (26.9%)	141 (25.2%)	0.514	348 (26.8%)	118 (24.8%)	118 (27.5%)	0.251
2019	400 (23.6%)	149 (26.6%)		311 (23.9%)	103 (21.7%)	118 (27.5%)	
2020	307 (18.1%)	96 (17.1%)		236 (18.2%)	92 (19.4%)	65 (15.2%)	
2021	535 (31.5%)	174 (31.1%)		404 (31.1%)	162 (34.1%)	128 (29.8%)	

Note: *P* values indicate the statistical significance of differences in participants’ characteristics between groups with and without a self-reported prior diagnosis of depressive disorder, and between groups based on the number of bad mental health days. Specifically, a one-way ANOVA was used to test differences in the age of diabetes diagnosis between groups. Chi-square tests were used to test differences in all other characteristics between groups.

The number of bad mental health days per month was associated with more diabetes management outcomes, including ever attending a diabetes management class, annual foot check by a professional, and annual dilated eye exam. Only 51.7% of those who reported 14 or more bad mental health days had taken a diabetes management class compared to 57.4% of participants who reported 1–13 bad mental health days and 59.0% of those who reported no bad mental health days (p = 0.032). Only 71.6% of participants who reported 14 + bad mental health days had completed an annual dilated eye exam, compared to 77.3% among those who reported 1–13 bad mental health days and 78.4% among those who reported no bad mental health days (p = 0.016). The percentage completing an annual dilated eye exam among those who had 14 + bad mental health days was 62.0%, lower than among those who had 13 + bad mental health days (77.3%) and those who had no bad mental health days (78.4%) (p < 0.001). Self-reported prior diagnosis of depressive disorder, age, sex, marital status, employment status, income, and age of diabetes diagnosis significantly differed by self-reported prior diagnosis of depressive disorders.

Table 3 displays the association between self-reported prior diagnosis of depressive disorder, number of bad mental health days, and diabetes management behaviors from the logistic regression using listwise deletion of missing values. Controlling for all covariates but not ever taking diabetes management class, depressive disorder was significantly associated with only foot self-checking at least once per day, among all diabetes management behaviors. Specifically, those who were diagnosed with depressive disorder had 44% lower odds to check their feet at least once per day than their counterparts who were not diagnosed with depressive disorder (AOR = 0.56; 95% CI: 0.34–0.92; p = 0.028). This association remained statistically significant even after adjusting for ever taking diabetes management class (AOR = 0.57; 95% CI: 0.34–0.94; p = 0.023). No significant difference was apparent for the odds of any diabetes management behavior between those who reported no bad mental health days and those who reported 1–13 bad mental health days per month after controlling for covariates. However, significant differences were evident for ever taking diabetes management class and foot self-check between those who reported no bad mental health days and those who reported at least 14 bad mental health days per month. Participants who reported at least 14 bad mental health days had 41% lower odds to have ever taken a diabetes management class than those who reported no bad mental health days (AOR = 0.59; 95% CI: 0.36–0.99; p = 0.046) controlling for covariates. Controlling for all covariates but not ever taking diabetes management class, those who had at least 14 bad mental health days per month had 73% lower odds to check their feet at least once per day than those who reported no bad mental health days (AOR = 0.37; 95% CI: 0.21–0.65; p < 0.001). This difference remained statistically significant after further adjusting for ever taking a diabetes management class (AOR = 0.38; 95% CI: 0.22–0.66; p < 0.001).

**Table 3:** Association between self-reported prior diagnosis of a depressive disorder, number of bad mental health days, and diabetes management behaviors from logistic regression models using listwise deletion of missing values.

		took diabetes management class	foot self-check	glucose self-check	doctor visit for diabetes	HbA_1c_ check	professional foot check	professional eye exam
***Models***	***Key predictors***	***AOR [95% CI]***	***AOR [95% CI]***	***AOR [95% CI]***	***AOR [95% CI]***	***AOR [95% CI]***	***AOR [95% CI]***	***AOR [95% CI]***
**1**	depressive disorder	0.67	0.56*	0.88	0.82	0.86	1.15	0.81
		[0.43, 1.04]	[0.34, 0.92]	[0.52, 1.51]	[0.50, 1.33]	[0.52, 1.32]	[0.68, 1.96]	[0.52, 1.26]
**2**	depressive disorder	NA	0.57*	0.94	0.84	0.91	1.22	0.82
			[0.34, 0.94]	[0.56, 1.58]	[0.51, 1.38]	[0.54, 1.53]	[0.72, 2.08]	[0.53, 1.28]
	took diabetes management class	NA	1.37	1.87**	1.61*	2.05**	2.13**	1.37
			[0.87, 1.99]	[1.21, 2.89]	[1.03, 2.52]	[1.31, 3.21]	[1.32, 3.43]	[0.89, 2.11]
**3**	bad mental health days (ref: none)							
	1-13 days	0.73	0.90	0.65	0.97	0.89	1.12	1.56
		[0.45, 1.20]	[0.54, 1.50]	[0.37, 1.16]	[0.56, 1.68]	[0.48, 1.64]	[0.63, 1.99]	[0.90, 2.70]
	14 + days	0.59*	0.37***	0.74	1.34	1.10	1.09	1.20
		[0.36, 0.99]	[0.21, 0.65]	[0.42, 1.32]	[0.77, 2.32]	[0.63, 1.91]	[0.62, 1.91]	[0.71, 2.04]
**4**	bad mental health days (ref: 0 day)	NA						
	1-13 days	NA	0.91	0.67	1.01	0.95	1.17	1.62
			[0.55, 1.52]	[0.38, 1.18]	[0.58, 1.74]	[0.51, 1.78]	[0.66, 2.73]	[0.95, 2.75]
	14 + days	NA	0.38***	0.80	1.41	1.19	1.17	1.25
			[0.22, 0.66]	[0.45, 1.39]	[0.81, 2.47]	[0.67, 2.10]	[0.67, 2.05]	[0.74, 2.13]
	took diabetes management class	NA	1.28	1.78**	1.59*	2.02**	2.00**	1.44
			[0.85, 1.93]	[1.14, 2.76]	[1.02, 2.50]	[1.29, 3.16]	[1.24, 3.22]	[0.93, 2.23]

Note: (1) AOR: adjusted odds-ratio; (2) All AORs were estimated after adjusting for demographic information, wave, health insurance, and access to health providers; (3) All AORs were population-level estimates obtained by applying sample weights; (4) *: *p* < 0.05, **: *p* < 0.01; ***: *p* < 0.001.

Table S1 in Supporting Information displays the association between self-reported prior diagnosis of depressive disorder, number of bad mental health days, and diabetes management behaviors from the logistic regression using multiple imputation by chained equations (MICE). While the association between self-reported prior diagnosis of depressive disorder and foot self-check was no longer statistically significant, the associations between number of bad mental health days per month, ever taking a diabetes management class, and foot self-check remained robust.

## Discussion

In general, this study underscores the importance of mental health in managing diabetes among AI/AN adults. The percentage of AI/AN participants who reported a prior diagnosis of depressive disorder in this study (25%) is consistent with previous research on this population. For example, Knaster and colleagues (2015), using Indian Health Service data, found that 965 out of 3,390 AI/AN adults with diabetes (28%) had a documented diagnosis of depression [33]. This prevalence is substantially higher than that reported among diabetes patients in the general U.S. population, which a meta-analysis estimated at 9.2% [34]. The consistently elevated prevalence of depressive disorder among AI/AN adults with diabetes compared to the general adult population highlights the need to strengthen mental health screening and support within diabetes care programs serving this population.

Results suggest that participants with a self-reported diagnosis of depressive disorder were less likely to check their feet daily than those without depressive disorders. The association between depressive disorder and other diabetes management behaviors was not statistically significant, however. Most studies that examined the association between mental health and diabetes management behaviors were conducted either outside the United States or were limited by small sample sizes. For example, two studies, including one in Iran and one in Germany, showed that depressive symptoms are significantly associated with lower self-management behavior, but did not examine the association for specific behaviors [35,36]. Examining data from 42 African American adults with Type 2 diabetes, Chlebowy and associates found that depression and anxiety were positively associated with HbA_1c_, but not with diet, physical exercise, or medical adherence [37]. To our knowledge, only two studies have examined the association between depression and diabetes management behaviors for the general U.S. adult population [27,38]; none focused on the association among AI/AN adults in the U.S. Importantly, the findings of this study differ from those reported in two similar studies that focused on the general U.S. population [27,38]. Specifically, the significant association between depressive disorder and foot self-check was *not* observed in the general U.S. population [27,38]. Moreover, we did not observe the significant associations between depressive disorder and diabetes management behaviors reported for the general U.S. population, i.e., lower adjusted odds of adherence to glucose self-check [27], professional foot check [27], and professional eye exam [27,38].

Several reasons may explain these discrepancies. First, the differential sample size between the two studies may contribute to differences in identified statistical significance. The previous two studies on the general U.S. population both had much larger sample sizes, over 7 and 50 times greater than the current study [27,38]. The much larger sample sizes in the two previous studies [27,38] may yield lower standard errors even though many estimated relationships in the current study had lower adjusted ORs. Second, the use of self-reported diagnosis for depressive disorder by a doctor may lack cultural sensitivity for AI/AN populations [39], introduce measurement errors, and weaken the observed associations. More specifically, for AI/AN ILDs, self-reported depression may be compounded by mental health stigma, historical trauma, and distrust toward the healthcare system, potentially hindering them from seeking mental health support, resulting in an underdiagnosis of depressive disorder [39,40]. This underestimation can lead to an incomplete understanding of its association with diabetes management behaviors for AI/AN ILDs. Consequently, the true relationships between depressive disorder and diabetes management behaviors among AI/AN ILDs may be at least as pronounced than those among the general U.S. population, contrary to our study’s findings

Participants who reported 14 or more bad mental health days were also less likely to take a diabetes management class and less likely to perform daily foot checks than those reporting no bad mental health days among the AI/AN ILDs. To our knowledge, only one prior study examined the association between bad mental health days and diabetes management behaviors among the U.S. adult population [27]. That study found significant associations between bad mental health days and lower adherence to self-foot checks, professional foot checks, and professional eye exams. However, those associations did not adjust for potential covariates, which may have confounded the effect of bad mental health days on diabetes management behaviors. By controlling for covariates, our study provided more robust evidence regarding the unique associations between bad mental health days and ever taking a diabetes management class or foot self-check for AI/AN ILDs.

These findings may have important implications for diabetes management strategies aimed at AI/AN adults. Given the observed associations between mental health and diabetes management behaviors, our results suggest that incorporating mental health support into diabetes care may help improve engagement in self-management practices. Diabetes management classes can integrate traditional foods and practices together with mental health education by involving community health representatives or elders who lead discussions on the interplay between mental health and diabetes care. Storytelling within these classes can convey personal experiences of successfully managing both mental health and diabetes, which could help foster motivation and a sense of community support. Specifically, a module on daily foot checks can be coupled with training on mindfulness and stress reduction techniques to support mental health, which may alleviate the negative impact of mental health on foot self-check.

The difference in findings between this AI/AN-focused study and previous research on the general U.S. population may indicate the value of tailoring diabetes management strategies to the specific needs of AI/AN adults. Demonstrations of foot care can include traditional practices, such as foot soaking with medicinal herbs, creating a holistic and arguably more acceptable approach consistent with AI/AN cultural practices and traditions. Additionally, establishing community-based and locally organized diabetes peer support groups may provide sustained resources for both mental well-being and adherence to diabetes self-management behaviors. These integrated strategies may help address mental health as a relevant component of diabetes care, potentially making self-management practices more accessible, acceptable, and meaningful for AI/AN adults.

This study focused on AI/AN adults with diabetes because of their disproportionate burden of diabetes and limited representation in previous research. We recognize that similar associations between mental health and diabetes management behaviors may exist in the general adult U.S. population or other racial/ethnic populations [27,37,38]. Our findings should not be interpreted as evidence of AI/AN-specific associations in the absence of comparative analysis. Instead, this study highlights the importance of considering mental health in diabetes management within AI/AN communities. In addition, to our knowledge, no research has explored whether the associations investigated in this study differ by race/ethnicity, which calls for future investigation to enhance the understanding of the relationship between mental health and diabetes management across diverse populations.

Several limitations should be acknowledged. Using self-reported data may introduce recall bias or social desirability bias, potentially leading participants to underreport the number of bad mental health days due to social stigma or overstate their adherence to diabetes management. Those biases may drive our estimates toward the null and reduce the likelihood of detecting statistically significant associations. Hence, the observed relationships in our analysis may be conservative estimates of the true effects. Furthermore, the findings of this study may not account for certain unmeasured confounders, such as cultural practices for mental health and diabetes and geographical differences, as well as potential residual confounding by latent variables not captured in the dataset, namely perceived discrimination, mental health stigma, food insecurity, or health system barriers, which may affect both mental health and diabetes management behaviors. Another limitation is the cross-sectional design that precludes a causal conclusion and fails to capture the potential bidirectional relationship between mental health and diabetes management. While our study focused on mental health as a predictor of diabetes management, it is also possible that daily burden, social stigma, and complications associated with diabetes management may contribute to depressive symptoms [41]. Future research should adopt diverse designs to effectively investigate the dynamics between mental health and diabetes management behaviors and strengthen causal inference. Quantitative research could use prospective cohort designs with repeated measures to rigorously assess temporal sequences of diabetes management behaviors and mental health, whereas qualitative approaches could be helpful to contextualize and validate the quantitative findings. In addition, although the trichotomization of bad mental health days per month (i.e., 0, 1–13, and 14 + days) is consistent with BRFSS coding and widely used in public health research to define “frequent mental distress,” it may obscure meaningful variation within groups. For example, individuals with 1 versus 13 days of distress are grouped together, despite potentially differing experiences. We chose this categorization due to the zero-inflated and right-skewed distribution of the variable and limited sample sizes within subgroups, which posed challenges for more granular categorization. Future research with larger and more balanced samples should explore alternative approaches, such as treating bad mental health days as a continuous variable or using non-linear modeling, to capture more nuanced mental health gradients. Finally, we did not adjust for multiple comparisons, as this was an exploratory study designed to generate hypotheses. Thus, findings should be interpreted with caution and confirmed in future studies with larger and more diverse samples.

Despite these limitations, our findings have important clinical and public health implications, particularly for the AI/AN population. Healthcare providers may consider integrating mental health screening and support into routine diabetes care—such as assessing depressive symptoms and emotional distress—as these may be linked to diabetes management behaviors in this population. Additionally, policy initiatives may benefit from supporting mental health resources within diabetes care systems that serve AI/AN adults. Increased support for diabetes management programs that integrate mental health and community-based practices may contribute to improved engagement in self-management among AI/AN adults with diabetes.

## Supporting information

Table S1 Association between self-reported prior diagnosis of a depressive disorder, number of bad mental health days, and diabetes management behaviors from logistic regression models using multiple imputation by chained equation. (DOCX)

## References

[1] Saeedi P, Petersohn I, Salpea P, Malanda B, Karuranga S, Unwin N, et al. Global and regional diabetes prevalence estimates for 2019 and projections for 2030 and 2045: Results from the International Diabetes Federation Diabetes Atlas, 9th edition. Diabetes Res Clin Pract. 2019;157:107843. doi: 10.1016/j.diabres.2019.107843 31518657

[2] American Diabetes Association. Statistics About Diabetes. 2022 [cited 2 Sep 2023]. https://diabetes.org/about-us/statistics/about-diabetes

[3] Bullock A, Sheff K, Hora I, Burrows NR, Benoit SR, Saydah SH, et al. Prevalence of diagnosed diabetes in American Indian and Alaska Native adults, 2006-2017. BMJ Open Diabetes Res Care. 2020;8(1):e001218. doi: 10.1136/bmjdrc-2020-001218 32312721 PMC7199144

[4] U.S. Health and Human Services Office of Minority Health. Diabetes and American Indians/Alaska Natives. 2021 [cited 12 Sep 2023]. https://minorityhealth.hhs.gov/omh/browse.aspx?lvl=4&lvlid=33

[5] Bullock A, Sheff K, Hora I, Burrows NR, Benoit SR, Saydah SH, et al. Prevalence of diagnosed diabetes in American Indian and Alaska Native adults, 2006-2017. BMJ Open Diabetes Res Care. 2020;8(1):e001218. doi: 10.1136/bmjdrc-2020-001218 32312721 PMC7199144

[6] Dai J, Chang J, Choi JM, Bullock A, Manson SM, O’Connell J, et al. Trends in anti-diabetic medication use, severe hyperglycaemia and severe hypoglycaemia among American Indian and Alaska Native Peoples, 2009-2013. Diabetes Obes Metab. 2025;27(1):328–37. doi: 10.1111/dom.16021 39434448 PMC11908821

[7] Burrows NR, Zhang Y, Hora I, Pavkov ME, Sheff K, Imperatore G, et al. Sustained Lower Incidence of Diabetes-Related End-Stage Kidney Disease Among American Indians and Alaska Natives, Blacks, and Hispanics in the U.S., 2000-2016. Diabetes Care. 2020;43(9):2090–7. doi: 10.2337/dc20-0495 32616609 PMC8628545

[8] Rietz M, Lehr A, Mino E, Lang A, Szczerba E, Schiemann T, et al. Physical Activity and Risk of Major Diabetes-Related Complications in Individuals With Diabetes: A Systematic Review and Meta-Analysis of Observational Studies. Diabetes Care. 2022;45(12):3101–11. doi: 10.2337/dc22-0886 36455117 PMC9862380

[9] Akhaury K, Chaware S. Relation Between Diabetes and Psychiatric Disorders. Cureus. 2022;14(10):e30733. doi: 10.7759/cureus.30733 36447711 PMC9699801

[10] Harding JL, Pavkov ME, Magliano DJ, Shaw JE, Gregg EW. Global trends in diabetes complications: a review of current evidence. Diabetologia. 2019;62(1):3–16. doi: 10.1007/s00125-018-4711-2 30171279

[11] Gregg EW, Sattar N, Ali MK. The changing face of diabetes complications. Lancet Diabetes Endocrinol. 2016;4(6):537–47. doi: 10.1016/S2213-8587(16)30010-9 27156051

[12] Cole JB, Florez JC. Genetics of diabetes mellitus and diabetes complications. Nat Rev Nephrol. 2020;16(7):377–90. doi: 10.1038/s41581-020-0278-5 32398868 PMC9639302

[13] Hayes A, Arima H, Woodward M, Chalmers J, Poulter N, Hamet P, et al. Changes in Quality of Life Associated with Complications of Diabetes: Results from the ADVANCE Study. Value Health. 2016;19(1):36–41. doi: 10.1016/j.jval.2015.10.010 26797234

[14] Garrett C, Doherty A. Diabetes and mental health. Clin Med (Lond). 2014;14(6):669–72. doi: 10.7861/clinmedicine.14-6-669 25468856 PMC4954143

[15] Diabetes Canada Clinical Practice Guidelines Expert Committee, Robinson DJ, Coons M, Haensel H, Vallis M, Yale J-F. Diabetes and Mental Health. Can J Diabetes. 2018;42 Suppl 1:S130–41. doi: 10.1016/j.jcjd.2017.10.031 29650085

[16] Trikkalinou A, Papazafiropoulou AK, Melidonis A. Type 2 diabetes and quality of life. World J Diabetes. 2017;8(4):120–9. doi: 10.4239/wjd.v8.i4.120 28465788 PMC5394731

[17] American College of Cardiology. Caught Between Two Worlds: Cardiovascular Care in American Indians and Alaska Natives. 2020 [cited 12 Sep 2023]. https://www.acc.org/Latest-in-Cardiology/Articles/2020/10/01/01/42/Cover-Story-Caught-Between-Two-Worlds-Cardiovascular-Care-in-American-Indians-and-Alaska-Natives

[18] Adakai M, Sandoval-Rosario M, Xu F, Aseret-Manygoats T, Allison M, Greenlund KJ, et al. Health Disparities Among American Indians/Alaska Natives - Arizona, 2017. MMWR Morb Mortal Wkly Rep. 2018;67(47):1314–8. doi: 10.15585/mmwr.mm6747a4 30496159 PMC6276383

[19] Kirwan JP, Sacks J, Nieuwoudt S. The essential role of exercise in the management of type 2 diabetes. Cleve Clin J Med. 2017;84(7 Suppl 1):S15–21. doi: 10.3949/ccjm.84.s1.03 28708479 PMC5846677

[20] Soto SC, Louie SY, Cherrington AL, Parada H, Horton LA, Ayala GX. An ecological perspective on diabetes self-care support, self-management behaviors, and hemoglobin A1C among Latinos. Diabetes Educ. 2015;41(2):214–23. doi: 10.1177/0145721715569078 25656696

[21] Kirk JK, Arcury TA, Ip E, Bell RA, Saldana S, Nguyen HT, et al. Diabetes symptoms and self-management behaviors in rural older adults. Diabetes Res Clin Pract. 2015;107(1):54–60. doi: 10.1016/j.diabres.2014.10.005 25467626 PMC4309740

[22] Kent D, D’Eramo Melkus G, Stuart PMW, McKoy JM, Urbanski P, Boren SA, et al. Reducing the risks of diabetes complications through diabetes self-management education and support. Popul Health Manag. 2013;16(2):74–81. doi: 10.1089/pop.2012.0020 23405872

[23] Rasoul AM, Jalali R, Abdi A, Salari N, Rahimi M, Mohammadi M. The effect of self-management education through weblogs on the quality of life of diabetic patients. BMC Med Inform Decis Mak. 2019;19(1):205. doi: 10.1186/s12911-019-0941-6 31665001 PMC6819410

[24] Scarton LJ, de Groot M. Emotional and Behavioral Aspects of Diabetes in American Indians/Alaska Natives. Health Educ Behav. 2017;44(1):70–82. doi: 10.1177/1090198116639289 27179289

[25] Kalra S, Jena BN, Yeravdekar R. Emotional and Psychological Needs of People with Diabetes. Indian J Endocrinol Metab. 2018;22(5):696–704. doi: 10.4103/ijem.IJEM_579_17 30294583 PMC6166557

[26] Centers for Disease Control and Prevention. Behavioral Risk Factor Surveillance System. 22 Nov 2024 [cited 24 Dec 2024]. https://www.cdc.gov/brfss/index.html

[27] Lai TC, McDaniel CC, Chou C. Diabetes management behaviors associated with depression in the U.S. Diabetol Metab Syndr. 2022;14(1):178. doi: 10.1186/s13098-022-00953-3 36419073 PMC9685969

[28] Centers for Disease Control and Prevention. 2021 BRFSS Survey Data and Documentation. 21 Jul 2023 [cited 24 Dec 2024]. https://www.cdc.gov/brfss/annual_data/annual_2021.html

[29] Cree RA, Okoro CA, Zack MM, Carbone E. Frequent Mental Distress Among Adults, by Disability Status, Disability Type, and Selected Characteristics - United States, 2018. MMWR Morb Mortal Wkly Rep. 2020;69(36):1238–43. doi: 10.15585/mmwr.mm6936a2 32914770 PMC7499832

[30] Link BG, Phelan J. Social Conditions As Fundamental Causes of Disease. Journal of Health and Social Behavior. 1995;35:80. doi: 10.2307/2626958 7560851

[31] Adams KF, Sperl-Hillen JM, Davis H, Spain CV, Hanson AM, Fernandes OD, et al. Factors Influencing Patient Completion of Diabetes Self-Management Education. Diabetes Spectrum. 2013;26(1):40–5. doi: 10.2337/diaspect.26.1.40

[32] Woods AD, Gerasimova D, Van Dusen B, Nissen J, Bainter S, Uzdavines A, et al. Best practices for addressing missing data through multiple imputation. Infant and Child Development. 2023;33(1). doi: 10.1002/icd.2407

[33] Knaster ES, Fretts AM, Phillips LE. The association of depression with diabetes management among urban American Indians/Alaska Natives in the United States, 2011. Ethn Dis. 2015;25(1):83–9. 25812257

[34] Wang F, Wang S, Zong Q-Q, Zhang Q, Ng CH, Ungvari GS, et al. Prevalence of comorbid major depressive disorder in Type 2 diabetes: a meta-analysis of comparative and epidemiological studies. Diabet Med. 2019;36(8):961–9. doi: 10.1111/dme.14042 31127631

[35] Azami G, Soh KL, Sazlina S-G, Salmiah MdS, Khosravi A, Aazami S, et al. The Effect of Depression on Poor Glycemic Control in Adults with Type 2 Diabetes: The Mediating Roles of Self-Efficacy and Self-Management Behaviors. Dubai Diabetes Endocrinol J. 2019;25(3–4):80–9. doi: 10.1159/000502126

[36] Schmitt A, Bendig E, Baumeister H, Hermanns N, Kulzer B. Associations of depression and diabetes distress with self-management behavior and glycemic control. Health Psychol. 2021;40(2):113–24. doi: 10.1037/hea0001037 33252963

[37] Chlebowy DO, Batscha C, Kubiak N, Crawford T. Relationships of Depression, Anxiety, and Stress with Adherence to Self-Management Behaviors and Diabetes Measures in African American Adults with Type 2 Diabetes. J Racial Ethn Health Disparities. 2019;6(1):71–6. doi: 10.1007/s40615-018-0500-3 29845520

[38] Egede LE, Ellis C, Grubaugh AL. The effect of depression on self-care behaviors and quality of care in a national sample of adults with diabetes. Gen Hosp Psychiatry. 2009;31(5):422–7. doi: 10.1016/j.genhosppsych.2009.06.007 19703635

[39] Beals J, Manson SM, Whitesell NR, Mitchell CM, Novins DK, Simpson S, et al. Prevalence of major depressive episode in two American Indian reservation populations: unexpected findings with a structured interview. Am J Psychiatry. 2005;162(9):1713–22. doi: 10.1176/appi.ajp.162.9.1713 16135632

[40] Stewart TJ, Gonzalez VM. Associations of historical trauma and racism with health care system distrust and mental health help-seeking propensity among American Indian and Alaska Native college students. Cultur Divers Ethnic Minor Psychol. 2023;29(3):348–57. doi: 10.1037/cdp0000587 37067492

[41] Tareen RS, Tareen K. Psychosocial aspects of diabetes management: dilemma of diabetes distress. Transl Pediatr. 2017;6(4):383–96. doi: 10.21037/tp.2017.10.04 29184819 PMC5682378

