# Supplementary material for: Depressive disorders, bad mental health days, and diabetes management behaviors among non-Hispanic American Indian/Alaska Native adults: Findings from the Behavioral Risk Factor Surveillance System
Source: PLoS One. 2025 Jul 14;20(7):e0327870. doi: 10.1371/journal.pone.0327870 (PMC12258579; doi:10.1371/journal.pone.0327870)
Supplement: Table S1 — (DOCX) [file pone.0327870.s001.docx]

Supporting Information

Table S1 Association between self-reported prior diagnosis of depressive disorder, number of bad mental health days, and diabetes management behaviors from logistic regression models using multiple imputation by chained equation

|  |  | **took diabetes management class** | **feet self-check** | **glucose self-check** | **doctor visit for diabetes** | **HbA_1c_ check** | **professional feet check** | **professional eye exam** |
| --- | --- | --- | --- | --- | --- | --- | --- | --- |
| ***Models*** | ***Key predictors*** | ***AOR***  ***[95% CI]*** | ***AOR***  ***[95% CI]*** | ***AOR***  ***[95% CI]*** | ***AOR***  ***[95% CI]*** | ***AOR***  ***[95% CI]*** | ***AOR***  ***[95% CI]*** | ***AOR***  ***[95% CI]*** |
| **1** | depressive disorder | 0.86 | 0.70 | 1.00 | 0.93 | 0.78 | 0/90 | 0.78 |
|  |  | [0.57, 1.29] | [0.44, 1.11] | [0.63, 1.58] | [0.51, 1.21] | [0.51, 1.21] | [0.57, 1.43] | [0.51, 1.18] |
| **2** | depressive disorder | NA | 0.71 | 1.03 | 0.95 | 0.80 | 0.94 | 0.79 |
|  |  |  | [0.44, 1.14] | [0.66, 1.60] | [0.61, 1.48] | [0.51, 1.26] | [0.59, 1.49] | [0.52, 1.19] |
|  | took diabetes management class | NA | 1.56* | 2.15*** | 1.81** | 2.04*** | 2.73*** | 1.69** |
|  |  |  | [1.08, 2.27] | [1.46, 3.18] | [1.22, 2.69] | [1.37, 3.05] | [1.79, 4.17] | [1.14, 2.48] |
| **3** | bad mental health days (ref: none) |  |  |  |  |  |  |  |
|  | 1-13 days | 0.82 | 0.72 | 0.83 | 1.10 | 0.88 | 0.93 | 1.65 |
|  |  | [0.52, 1.31] | [0.45, 1.14] | [0.51, 1.35] | [0.68, 1.80] | [0.53, 1.47] | [0.57, 1.53] | [0.96, 2.84] |
|  | 14+ days | 0.56* | 0.48** | 0.84 | 1.25 | 0.96 | 0.86 | 0.88 |
|  |  | [0.35, 0.90] | [0.30, 0.79] | [0.50, 1.40] | [0.75, 2.07] | [0.59, 1.53] | [0.51, 1.44] | [0.56, 1.39] |
| **4** | bad mental health days (ref: 0 day) | NA |  |  |  |  |  |  |
|  | 1-13 days | NA | 0.73 | 0.86 | 1.15 | 0.91 | 0.97 | 1.70* |
|  |  |  | [0.46, 1.17] | [0.53, 1.38] | [0.71, 1.85] | [0.54, 1.53] | [0.59, 1.59] | [1.01, 2.88] |
|  | 14+ days | NA | 0.50** | 0.94 | 1.36 | 1.04 | 0.98 | 0.94 |
|  |  |  | [0.31, 0.83] | [0.57, 1.53] | [0.83, 2.25] | [0.64, 1.70] | [0.59, 1.65] | [0.59, 1.48] |
|  | took diabetes management class | NA | 1.50* | 2.14*** | 1.87** | 2.05*** | 2.73*** | 1.70** |
|  |  |  | [1.03, 2.16] | [1.45, 3.15] | [1.26, 2.76] | [1.37, 3.08] | [1.79, 4.16] | [1.16, 2.49] |

Note: (1) AOR: adjusted odds-ratio; (2) All AORs were estimated after adjusting for demographic information, wave, health insurance, and access to health providers; (3) All AORs were population-level estimates obtained by applying sample weights; (4) *: *p* < 0.05, **: *p* < 0.01; ***: *p* < 0.001.
